# HCV RNA detection in hepatic and extrahepatic tissues after DAA-induced sustained virologic response

**DOI:** 10.17305/bb.2026.13329

**Published:** 2026-06-14

**Authors:** Samah Mamdouh, Hussam Hamdy, Tarek Aboushousha, Mona Magdy, Gehan Hammad, Mohamed Shemis, Amr Abdelraouf

**Affiliations:** 1 Department of Biochemistry and Molecular Biology, Theodor Bilharz Research Institute, Giza, Egypt; 2 Department of General Surgery, Theodor Bilharz Research Institute, Giza, Egypt; 3 Department of Pathology, Theodor Bilharz Research Institute, Giza, Egypt; 4 Faculty of Biotechnology, October University for Modern Sciences and Arts (MSA), Giza, Egypt; 5 Department of Hepato-Bilio-Pancreatic (HBP) Surgery, National Hepatology and Tropical Medicine Research Institute (NHTMRI), Cairo, Egypt

**Keywords:** Hepatitis C virus, HCV, direct-acting antivirals, DAAs, Sovaldi, sofosbuvir, sustained virologic response, SVR, strand-specific PCR.

## Abstract

The complete eradication of hepatitis C virus (HCV) from both hepatic and extrahepatic compartments following successful direct-acting antiviral (DAA) therapy remains an area of ongoing investigation. This study aimed to detect HCV RNA signals in serum and tissue samples from 40 patients who had achieved a sustained virologic response (SVR). Paired serum samples (pre-treatment and post-SVR) were analyzed concurrently with liver, gallbladder, bile, pancreatic, and splenic tissues. These tissue samples were obtained during clinically indicated surgical procedures performed 4–12 weeks after SVR. HCV RNA was quantified using a real-time PCR assay, adapted exploratorily for tissue matrices. Statistical analysis encompassed paired comparisons for serum samples and descriptive analysis for tissue findings. All patients exhibited complete clearance of serum HCV RNA post-treatment (*P* < 0.001). Nevertheless, HCV RNA signals were detected in 11 of 40 liver tissue samples (27.5%) and in 6 of 40 gallbladder and bile samples (15%). Furthermore, detection was observed in the sole pancreatic sample (1/1) and in two of three splenic samples (2/3). Due to the constrained sample sizes, the extrahepatic findings are presented descriptively. Viral load measurements varied across tissues and are reported as median values with interquartile ranges. In conclusion, HCV RNA signals were detected in hepatic and selected extrahepatic tissues despite undetectable serum PCR following DAA therapy. It is crucial to note that these findings represent molecular detection exclusively and do not confirm active viral replication or persistence. These results should be considered exploratory and necessitate validation through strand-specific assays and larger patient cohorts.

## Introduction

Hepatitis C virus (HCV), a single-stranded RNA virus, is a primary cause of liver inflammation and hepatocellular carcinoma (HCC). Transmission primarily occurs through contaminated blood, often via unsafe medical practices such as needle reuse or unscreened blood transfusions [1]. The World Health Organization (WHO) estimates that approximately 50 million people worldwide are chronically infected with HCV, with an estimated 1 million new infections annually. In 2022, HCV-related complications, predominantly cirrhosis and HCC, resulted in approximately 242,000 deaths. Egypt continues to report one of the highest HCV prevalence rates globally [2–4].

HCV, a member of the *Flaviviridae* family, possesses a 9.6 kb genome encoding a single open reading frame (ORF) that translates into a polyprotein of approximately 3,000 amino acids [1]. Upon entry into hepatocytes, the viral genome is released into the cytoplasm, where replication and translation synthesize viral proteins [5].

Despite the absence of an HCV vaccine, advancements in understanding the virus’s genome and protein structure have significantly improved treatment strategies. The advent of direct-acting antivirals (DAAs), which target nonstructural proteins to interfere with specific stages of the HCV life cycle, has revolutionized treatment outcomes [6, 7].

Current WHO guidelines recommend regimens including sofosbuvir, daclatasvir, and the sofosbuvir/ledipasvir combination, all demonstrating cure rates exceeding 95% [8, 9]. These DAAs offer substantial advantages in efficacy, safety, and tolerability compared to older therapies. Typically, a 12-week course of DAA therapy is sufficient for infection eradication [10]. However, pegylated interferon and ribavirin may still be considered in select cases where DAA-only regimens are inadequate [11].

Sofosbuvir has emerged as a groundbreaking therapy due to its pan-genotypic activity, once-daily dosing, lack of food restrictions, minimal side effects, limited drug interactions, high genetic barrier to resistance, and excellent efficacy in patients with advanced liver disease [12, 13].

Successful HCV therapy offers numerous benefits, including reduced hepatic fibrosis, lower risk of liver failure and HCC, increased survival, and improvement in some extrahepatic manifestations [14]. The benchmark for treatment success is a sustained virologic response (SVR), defined as undetectable HCV RNA six months post-treatment [15].

While SVR is generally considered indicative of viral eradication, a remote possibility of low-level viral persistence and subsequent reactivation cannot be entirely excluded [16–18]. Reinfection risk is low unless an individual is re-exposed to HCV, as antibodies formed during initial infection do not confer long-term immunity [19].

In Egypt, HCV has historically presented a significant public health challenge. Since 2006, the Ministry of Health, through the National Committee for Control of Viral Hepatitis (NCCVH), has prioritized treatment. A large-scale treatment program utilizing pegylated interferon and ribavirin was implemented between 2007 and 2015 [15, 20]. Egypt also pioneered a government-supported DAA-based treatment program [21], screening nearly 60 million people and treating approximately 3.5 million, making it the largest HCV elimination campaign globally.

This study aimed to investigate the potential for detectable HCV RNA post-treatment, confirm whether negative serum polymerase chain reaction (PCR) results indicate complete viral clearance, and establish a simple, real-time quantitative reverse transcription polymerase chain reaction (qRT-PCR) method for quantifying HCV RNA in serum, bile, and gallbladder tissue.

## Materials and methods

### Study design

This longitudinal study enrolled 40 adult patients (≥18 years) with chronic HCV infection. All patients received DAA therapy according to international guidelines, specifically sofosbuvir (400 mg/day) and daclatasvir (60 mg/day) for 12 weeks. During therapy, patients underwent clinical and laboratory assessments at weeks 2, 4, 8, and 12, including complete blood count (CBC), serum creatinine, albumin, alpha-fetoprotein (AFP), alanine aminotransferase (ALT), aspartate aminotransferase (AST), and total bilirubin.

All patients achieved a SVR, defined as undetectable serum HCV RNA at the end of treatment and six months thereafter, and were included in the study. Exclusion criteria comprised age <18 or >90 years, pregnancy or inability to use effective contraception, hepatic malignancy, inadequately controlled diabetes mellitus or hypertension, collagen vascular diseases, HCV-related chronic kidney disease, and co-infection with hepatitis B virus (HBV) or human immunodeficiency virus (HIV).

Surgical procedures were performed for independent clinical indications at the Departments of Surgery, Theodor Bilharz Research Institute and National Hepatology and Tropical Medicine Research Institute (Egypt). All patients underwent laparoscopic cholecystectomy, during which liver, gallbladder, and bile samples were obtained. Additionally, one patient underwent pancreatic tissue sampling, and three patients underwent splenectomy, providing splenic tissue samples.

All tissue samples were collected 4–12 weeks after SVR confirmation. To exclude reinfection, serum HCV RNA testing was repeated immediately prior to surgery and confirmed to be negative in all patients. Baseline histopathological data were retrieved from pre-treatment biopsies documented in patient medical records.

### Data collection

For all patients, demographic information, presence of liver cirrhosis, HCC, HIV coinfection, history of liver transplantation, interferon (IFN) treatment experience, and the type and duration of the treatment regimen were collected.

Patients underwent comprehensive history taking, clinical examination, and laboratory investigations, including CBC, ALT, AST, serum bilirubin, serum albumin, international normalized ratio (INR), AFP, serum creatinine, and abdominal ultrasonography, both before and after treatment.

### Biological samples

All 40 patients provided blood, liver, gallbladder, and bile specimens. One patient provided these specimens in addition to a pancreas tissue specimen, while three patients provided spleen tissue specimens. Blood samples were collected from all patients, centrifuged, and stored at −20^∘^C until analysis. A portion of the tissue specimens was prepared for histological examination, and the remaining portion was immediately frozen post-operatively.

### Surgical techniques

Laparoscopic cholecystectomy was performed using the standard 4-trocar technique under general anesthesia in nearly all cases. An open insufflation technique was employed to introduce a 10 mm trocar through the umbilicus, establishing a pneumoperitoneum (using CO_2_) at approximately 14 mmHg. The gallbladder was identified, carefully dissected, and the cystic duct and artery were separately dissected and clipped. The gallbladder was then placed in an endo-bag and extracted through the 10 mm epigastric port. Biopsies were performed 4–12 weeks after SVR confirmation. All patients underwent repeat serum HCV RNA testing within one week prior to surgery to rule out reinfection. For liver biopsies, each patient underwent a laparoscopically guided needle biopsy, and a standard 1 × 1 cm wedge biopsy from the liver was obtained via scalpel dissection, followed by either mono- or bipolar diathermy coagulation to ensure adequate hemostasis at the biopsy site. Trocar sites were closed after routine placement of a drain for approximately 12 h postoperatively. The postoperative period was standard, and patients were discharged on the first postoperative day after drain removal, with a follow-up visit one week later. All patients experienced favorable postoperative recovery. For each extracted gallbladder, bile was aspirated *in vitro* by fine needle puncture under complete aseptic conditions, adhering to infection control regulations. Wedge biopsies were obtained when sufficient hepatic tissue was available during elective laparoscopic procedures, while fine-needle biopsies (FNB) were utilized when minimal sampling was preferred to reduce procedural risk, such as coagulopathy or limited tissue access. This dual approach ensured both adequate tissue for histopathology and molecular analysis while maintaining patient safety. In our study, one case involved a pancreatic tissue sample and another involved a spleen tissue sample.

### Pancreatic tissue cases

A patient presented with a cystic lesion in the pancreatic head, preoperatively diagnosed as a mucinous cystic lesion. Consequently, a pancreaticoduodenectomy (Whipple’s resection) was performed using the traditional open technique. During the procedure, a biopsy of healthy pancreatic tissue was obtained from a clear zone of the resected specimen and subsequently included in the study, adhering to established protocols.

### Splenic tissue cases

Three cases involving splenic tissue were included. One patient underwent laparoscopic splenectomy for immune thrombocytopenic purpura (ITP) using the classical four-port technique. The spleen was retrieved via an endobag, and a 2 × 2 cm splenic tissue biopsy was obtained with a scalpel for inclusion in the study. The remaining two cases involved traditional open laparotomy splenectomy for road traffic accident (RTA) injuries. In these instances, a 2 × 2 cm splenic tissue biopsy was similarly taken with a scalpel and incorporated into the study.

### Tissue histology

Liver tissue biopsies were fixed in 10% formalin for 48 h, followed by processing through ascending concentrations of ethanol. Tissues were then cleared in xylene and infiltrated with melted wax. Finally, liver tissue specimens were embedded in paraffin blocks and sectioned at 5 µm thickness. For staining, tissue sections were de-paraffinized, rehydrated, and stained with Hematoxylin & Eosin (H&E) to evaluate histopathological changes. Stained sections were independently examined by two histopathologists under an Axio research microscope (Zeiss) equipped with a digital camera (MRC5, Zeiss) to assess the extent of liver damage.

Histopathological parameters considered during liver section examination included hepatitis activity, fibrosis (Metavir score), steatosis, and the presence of hepatocellular dysplasia and carcinoma [3].

### Virological determination

Approximately 150 mg of each tissue sample was homogenized in 1 mL RLT lysis buffer (Qiagen) containing guanidinium thiocyanate and β-mercaptoethanol. RNA integrity (A260/280 ≥ 1.8) was confirmed spectrophotometrically. To minimize contamination, RNA extraction, reverse transcription polymerase chain reaction (RT-PCR) setup, and amplification were conducted in physically separated rooms utilizing negative extraction controls and filter tips. HCV RNA levels were quantified using the Abbott Real-Time HCV Assay, which has a lower limit of detection (LLOD) of 12 IU/mL (Abbott Molecular System, RealTime™ HCV, Abbott Molecular Inc, Des Plaines, IL, USA). Although validated for serum, the Abbott RealTime HCV assay was employed in an exploratory manner for tissue samples. Spike-recovery and inhibition testing were performed in liver and bile matrices to assess assay performance. Viral loads are reported as copies per µg total RNA. All RNA extractions and qRT-PCR reactions were performed in separate, designated clean rooms using aerosol-resistant filter tips. Negative extraction controls were included with each batch, and all positive detections were confirmed in duplicate. While the Abbott RealTime assay is optimized for serum RNA, it was used here, with strict contamination control, to detect tissue-associated viral RNA at low copy levels.

### Ethical statement

The study received approval from the Ethics Committee of the National Hepatology and Tropical Medicine Research Institute (NHMRI, Egypt) under serial number: 0031012017. All participants provided written informed consent for the use of their specimens for research purposes, in accordance with institutional guidelines. All experimental procedures adhered to the latest version of the Declaration of Helsinki and general guidelines for good clinical practice. Clinical Trial Number: N/A.

### Statistical analysis

Data were analyzed using Microsoft Excel 365 and IBM SPSS Statistics, version 29.0.1.1.1 (IBM Corp., Armonk, N.Y., USA). Normally distributed continuous variables were expressed as mean ± standard deviation (SD). Non-parametrically distributed variables were represented as median and interquartile range (IQR, 25%–75%), with a 95% confidence interval. Categorical variables were summarized using frequencies and percentages. A *P* value < 0.05 was considered statistically significant. Continuous paired variables were analyzed using either a paired *t*-test or Wilcoxon signed-rank test. Paired categorical variables were analyzed using McNemar’s exact test. HCV RNA concentrations were log_1__0_-transformed. Non-detectable values were assigned LOD/2. Triplicate PCR values were averaged per patient. Analyses for pancreas (*n* ═ 1) and spleen (*n* ═ 3) were descriptive only due to small sample sizes.

## Results

### Demographic data and comparison of laboratory investigations before and after treatment

The overall mean patient age was 54.7 ± 9.9 years. The cohort comprised 16 females (40%) and 24 males (60%). Paired *t*-tests were used to compare paired parameters such as ALT, AST, and creatinine, while the Wilcoxon test was applied to compare total bilirubin and AFP. A highly statistically significant reduction was observed after treatment in hemoglobin (HB), INR, blood platelets, ALT, AST, total bilirubin, creatinine, and AFP (*P* < 0.001). Conversely, a highly statistically significant increase in albumin levels was noted after treatment (*P* < 0.001) (Table 1).

**Table 1:** Demographic and laboratory characteristics of the study population

		**Before treatment**	**After treatment**	***P* value**
Age		54.7 ± 9.9	–	–
Sex	Female	16 (40%)	–	–
	Male	24 (60%)	–	–
Hb (g/dl)		13.3 ± 2.3	12.34 ± 1.41	<0.001**
INR		1.2 ± 0.1	1.10 ± 0.09	<0.001**
PLAT (10^3^/cm)	260.3 ± 142.3	174.7 ± 66.99	<0.001**	
ALT (IU/ml)	41.0 ± 28.4	14.5 ± 3.9	<0.001**	
AST (IU/ml)	37.5 ± 22.4	17.7 ± 3.8	<0.001**	
Albumin (g/dl)	3.21 ± 0.47	3.99 ± 0.51	<0.001**	
Total bilirubin (mg/dl)	0.8 (0.6–1.1)	0.5 (0.4–0.6)	<0.001**	
Creatinine (mg/dl)	1.2 ± 0.4	0.8 ± 0.2	<0.001**	
AFP (ng/ml)	8.4 (5.6–31.2)	4.2 (3.4–5.4)	<0.001**	

Age, Hb, INR, PLAT, ALT, AST, albumin and creatinine are presented as mean ± SD. These data were analyzed using a paired *t*-test to compare the paired parameters. Sex is represented as frequency and percentage (F%). Bilirubin and AFP are presented as median and IQR. These data were analyzed using a Wilcoxon test to compare the paired parameters. *A* *P value of <0.05 was considered statistically *significant,* and a **P value of <0.01 was considered highly significant. **Abbreviations:** AFP, alpha-fetoprotein; ALT, alanine aminotransferase; AST, aspartate aminotransferase; F%, frequency and percentage; Hb, hemoglobin; INR, international normalized ratio; IQR, interquartile range; PLAT, platelet count; SD, standard deviation.

### Histopathological findings before and after treatment

After treatment, cases with grade 2 and 3 histological activity index (HAI) were no longer observed, and the number of cases with grade 1 HAI decreased. Similarly, the number of cases with steatosis scores 2 and 3 decreased, shifting towards scores 0 and 1. Regarding liver fibrosis, an apparent decrease in the percentage of cases previously categorized as stage 2, 3, and 4 was noted, suggesting a shift to stages 0 and 1. Post-therapy, a few new cases (3) of focal liver cell dysplasia and HCC were detected, which were undetectable before treatment (it is important to note that dysplasia and malignancy are contraindications for treatment) (Table 2).

**Table 2:** Comparison of histopathology and abdominal ultrasonography results before and after treatment

**Group**	**Point of comparison**	**Before treatment**	**After treatment**
	**0**	2 (5%)	28 (70%)
**Hepatitis activity**	**1**	20 (50%)	12 (30%)
	**2**	13 (32.5%)	-
	**3**	5 (12.5%)	-
	**0**	3 (7.5%)	17 (42.5%)
**Hepatic steatosis**	**1**	8 (20%)	14 (35%)
	**2**	14 (35%)	7 (17.5%)
	**3**	15 (37.5%)	2 (5%)
	**0**	3 (7.5%)	4 (10%)
	**1**	10 (25%)	16 (40%)
**Liver fibrosis**	**2**	16 (40%)	13 (32.5%)
	**3**	5 (12.5%)	3 (7.5%)
	**4**	6 (15%)	4 (10%)
**Liver cell dysplasia/HCC**	**Negative**	40 (100%)	37 (92.5%)
	**Positive**	0 (0.0%)	3 (7.5%)
	**Normal study**	5 (12.5%)	18 (45%)
	**Chronic parenchymatous**	14 (35%)	10 (25%)
**Ultrasonography**	**Bright liver**	0 (0.0%)	9 (22.5%)
	**Fatty hepatomegaly**	14 (35%)	0 (0.0%)
	**Cirrhotic liver**	7 (17.5%)	0 (0.0%)
	**HCC**	0 (0.0%)	3 (7.5%)

Histopathological and ultrasonographic data are presented as frequencies and percentages. Data are described descriptively; no inferential statistical testing is performed. **Abbreviation:** HCC, hepatocellular carcinoma.

### Abdominal ultrasonography findings before and after treatment

Descriptive changes observed in abdominal ultrasonography findings before and after treatment indicated liver improvement. These included an increase in the number of patients diagnosed with a normal liver study, a decrease in patients with chronic parenchymatous liver disease, and the emergence of 9 patients with a “bright liver” post-treatment, a diagnosis absent before therapy. Additionally, a decrease in the percentage of patients with fatty hepatomegaly was observed. Three patients (7.5%) developed *de novo* HCC, attributed to reasons unrelated to the treatment regimen (Table 2).

### PCR HCV before and after treatment

A total of 40 patients with chronic hepatitis C virus infection completed 12 weeks of DAA treatment. RNA signals were detected in 11/40 (27.5%) liver samples and 6/40 (15%) gallbladder or bile samples. Detection was also observed in the single pancreatic sample (1/1) and two of three splenic samples (2/3); these findings are presented descriptively due to the small sample sizes.

Six months post-treatment, all patients achieved a SVR with seronegative PCR for HCV RNA, maintaining undetectable serum HCV RNA levels.

HCV RNA measurements are presented as median and IQR (25th–75th percentile). Paired comparisons of serum HCV RNA before and after treatment were performed using the Wilcoxon signed-rank test for continuous values and McNemar’s exact test for paired categorical outcomes.

As shown in Table 3, median serum HCV RNA levels significantly decreased from 2.6 × 10^5^ (IQR: 1.5–6.5 × 10^5^) before treatment to undetectable levels after SVR (*P <* 0.001). Consequently, serum HCV RNA positivity declined from 100% (40/40) pre-treatment to 0% (0/40) post-treatment (*P <* 0.001).

**Table 3:** Detection of HCV RNA in serum and tissue samples following direct-acting antiviral therapy

**Sample type**	**n analyzed**	**HCV RNA positive, n (%)**	**95% CI (exact binomial)**	Median viral load* (copies/µg total RNA) (IQR)	**Statistical test**
Serum (before treatment)	40	40 (100%)	91.2–100%	2.6 ×10^5^ (1.5–6.5 ×10^5^)	—
Serum (after SVR)	40	0 (0%)	0–8.8%	Not detectable	McNemar’s test, *P* < 0.001
Liver tissue	40	11 (27.5%)	14.6–43.9%	17.9 ×10^5^ (11.4–35.8 ×10^5^)	Descriptive
Gallbladder tissue	40	6 (15%)	5.7–29.8%	15.6 ×10^5^ (13.5–17.5 ×10^5^)	Descriptive
Bile	40	6 (15%)	5.7–29.8%	20.3 ×10^5^ (16.2–28.8 ×10^5^)	Descriptive
Pancreatic tissue	1	1 (100%)	N/A	N/A	Descriptive
Splenic tissue	3	2 (66.7%)	N/A	N/A	Descriptive

A total of 40 paired serum samples (pre-treatment and sustained virologic response), 40 liver tissues, 40 gallbladder/bile samples, one pancreatic tissue sample, and three splenic tissue samples were analyzed. Inferential statistical testing was exclusively performed on paired serum samples utilizing McNemar’s exact test. Tissue data and findings from small sample sizes (pancreas, spleen) are presented descriptively. Viral load values are reported for positive samples only. **Abbreviations:** CI, confidence interval; HCV, hepatitis C virus; IQR, interquartile range; N/A, not applicable; RNA, ribonucleic acid; SVR, sustained virologic response.

HCV RNA signals were detected in 11 of 40 liver tissue samples (27.5%), with a median viral load of 17.9 × 10^5^ (IQR: 11.4–35.8 × 10^5^). Additionally, 6 of 40 patients (15%) showed detectable HCV RNA in both gallbladder tissue and bile samples, with median values of 15.6 × 10^5^ (IQR: 13.5–17.5 × 10^5^) and 20.3 × 10^5^ (IQR: 16.2–28.8 × 10^5^), respectively.

HCV RNA was also detected in the single pancreatic sample (1/1) and in two of three splenic tissue samples (2/3). Due to the limited sample size for these tissues, these findings are presented descriptively without inferential statistical testing.

## Discussion

This study evaluated the detection of HCV RNA signals in hepatic and extrahepatic tissues following SVR. While all patients achieved serum-negative status, HCV RNA signals were detected in a subset of tissues. The present work observed a statistically significant reduction in ALT, AST, total bilirubin, creatinine, platelet counts, and AFP after treatment. This aligns with previous research, specifically [22], which reported improved liver function within two years of DAA treatment initiation in UK patients, particularly those achieving SVR. Similarly, [23] noted a significant decline in ALT and AST. These findings underscore the significant role of DAAs in ameliorating hepatic necro-inflammatory changes induced by viral infection.

Contrary to some reports, such as [24, 25], which indicated a decrease in platelet count immediately after DAA treatment in chronic hepatitis C patients, our study found a significant decrease in platelet count from 260.3 ± 142.3 to 174.7 ± 66.99 after DAA therapy. This reduction is attributed to a decrease in HCV-induced platelet-associated immunoglobulin G levels. However, [26] did not observe significant changes in platelet count before and after treatment.

In our study, a significant difference was observed in pre-and post-treatment hemoglobin levels, which contradicts the findings of [26], who reported no significant difference.

Histological follow-up of patients’ livers revealed an improvement in the HAI and liver fibrosis. Specifically, cases with grade 2 and 3 HAI were not observed post-treatment, and the number of cases with grade 1 HAI decreased. These findings are consistent with [25], who reported that 71.4% of patients experienced improved necroinflammation HAI scores and a significant decrease in mean HAI scores after antiviral therapy.

Regarding the histopathological assessment of liver fibrosis, we observed a decrease in the percentage of cases previously categorized as stage 2, 3, and 4, suggesting a shift to stages 0 and 1. These results are supported by [27], who noted significant liver fibrosis regression after HCV therapy, and by [28], who found that DAA treatment for chronic HCV is associated with regression of liver fibrosis, with approximately 28% of patients improving from severe fibrosis (≥F3) to non-significant fibrosis (≤F2) after treatment.

Our data indicated an apparent decrease in the number of cases with score 2 and 3 steatosis post-treatment, shifting to scores 0 and 1. This finding diverges from [27], who observed a resolution of liver fat in only 11.1% of patients with baseline steatosis, while the remaining cases showed persistent or increased steatosis. Additionally, clinical fatty liver was detected in 20% of patients without baseline steatosis. More recently, Truscello et al. (2025) reported that the rate of change for the hepatitis steatosis index (HSI) remained relatively stable before and after treatment, which contradicts our results [29].

Regarding focal liver cell dysplasia and HCC after therapy, we recorded three (7.5%) emerging cases that were undetectable before treatment. This emergence is attributed to risk factors such as older age, male gender, advanced liver fibrosis, and findings from liver CT. Previous studies have presented conflicting results regarding the effect of DAAs on HCC development or recurrence. Unlike some studies that raised concerns about DAA therapy increasing or accelerating the risk of HCC development or recurrence and leading to more aggressive tumors, our study’s results differ. We observed de novo HCC only in cirrhotic patients, suggesting that underlying liver disease, rather than DAA therapy, is the primary risk factor. This contrasts with [30], who recorded 12.22% of patients developing new HCC during follow-up. Comparatively, some studies reported lower rates than ours, such as [31], who found that HCC developed in 5.4% of patients during the study period. Although three cases of de novo HCC occurred during follow-up, their tissues were not analyzed for residual HCV RNA; therefore, no causal relationship between viral residues and hepatocarcinogenesis can be inferred. The findings support that cirrhosis—not DAA therapy—is the principal risk factor for HCC after SVR.

The risk of HCC occurrence or recurrence after SVR with antiviral drug therapy remains unclear. However, [32] and co-authors reported that post-DAA HCC was diagnosed as a single nodule in 49% of cases, two nodules in 40%, and multifocal or diffuse infiltration in 11%. Furthermore, they concluded that post-DAA HCC was characterized by larger tumor diameter, a more diffuse pattern, a higher percentage of malignant portal vein thrombosis, and later Barcelona Clinic Liver Cancer (BCLC) stages at diagnosis. Nevertheless, the current study’s findings demonstrated that liver cirrhosis, particularly in Egyptian patients, is the cause of HCC development. Of the seven patients enrolled with liver cirrhosis, four experienced improvement; however, the remaining three cirrhotic patients unfortunately developed HCC. While no patients were categorized as cirrhotic on post-treatment ultrasound, imaging findings alone cannot confirm fibrosis regression. Due to their favorable safety and and efficacy, DAAs have significantly increased SVR rates in chronic HCV patients in recent years [19, 33].

This study aimed to demonstrate the presence of HCV RNA in liver tissue samples from 11 patients following treatment. It also indicated the prevalence of HCV in extrahepatic tissues, as it was found in the bile of six patients and in gallbladder tissues. Remarkably, HCV was detected in the spleen tissues of two patients and the pancreas of one patient. While our findings demonstrate the presence of viral RNA in hepatic and extrahepatic tissues, no cases of clinical reinfection were observed during follow-up. Thus, any link between residual RNA and future reinfection remains hypothetical and requires prospective monitoring. These findings are corroborated by Forton et al. [34], who collected tissue samples from the liver, brain (basal ganglia), serum, and, in one case, mesenteric lymph nodes within 36 h of the deaths of three patients who succumbed to complications related to cirrhosis caused by HCV infection. They observed that brain samples from two of the three patients contained HCV RNA, with the investigation conducted with institutional ethical committee consent. Our results are also consistent with those reported by [24], who discussed how in situ hybridization (ISH) and immunohistochemistry were used to identify HCV RNA and HCV antigens in the kidney, heart, pancreas, gut, adrenal gland, lymph node, and gallbladder. They concluded that HCV might infect extrahepatic tissues and that a wide variety of tissue cells could facilitate HCV replication when ISH revealed HCV RNA in bile duct epithelial cells, sinusoidal cells, and mononuclear cells in liver tissues. In summary, HCV RNA signals were detected in hepatic and selected extrahepatic tissues despite negative serum PCR following DAA therapy. These findings represent molecular detection only and do not establish viral replication. The results are exploratory and require validation in larger studies using strand-specific methods. The study underscores the need for larger, strand-specific investigations to clarify the biological significance of these residual viral traces. Long-term surveillance remains crucial, especially for cirrhotic patients who remain at elevated risk for HCC independent of DAA therapy. HCV RNA traces can persist in hepatic and extrahepatic tissues after successful DAA therapy, even when serum PCR is negative. While this detection of residual HCV RNA does not confirm active replication, it underscores the importance of strand-specific validation and long-term monitoring in patients with advanced liver disease.

### Study limitations

The present study is limited by its relatively small cohort size and the variability in the availability of extrahepatic tissue samples. The single-center design and absence of strand-specific RNA quantification limit the ability to confirm active replication vs residual RNA. Despite strict aseptic and contamination control procedures, trace contamination cannot be fully excluded. A formal validation of the Abbott RealTime assay for tissue matrices, including tissue-specific detection limits and recovery percentages, was not performed; therefore, these tissue findings are strictly exploratory. Future studies incorporating strand-specific assays, larger sample sizes, and multicentric validation are needed. Strand-specific assays to distinguish positive- and negative-strand HCV RNA were not performed, representing a limitation in confirming replication-competent virus. This will be addressed in future investigations using strand-specific RT-PCR.

## Conclusion

In conclusion, this study demonstrates that achieving a SVR and negative serum PCR does not guarantee complete viral clearance across all tissue compartments. The detection of residual HCV RNA in 27.5% of liver samples and 15% of gallbladder and bile samples, alongside traces in pancreatic and splenic tissues, indicates a disparity between systemic clearance and tissue-level persistence. As these exploratory findings represent molecular detection rather than confirmed active viral replication, validation with strand-specific assays in larger cohorts is essential. Ultimately, these results underscore the clinical necessity for ongoing post-SVR surveillance and long-term monitoring, particularly in patients with advanced liver cirrhosis who remain at an elevated risk for developing *de novo* hepatocellular carcinoma despite successful direct-acting antiviral therapy.

## Supplemental data

### Highlights

- HCV RNA was detected in the liver tissues of 11 patients and in extrahepatic tissues (gallbladder, bile, pancreas, spleen), even when serum PCR results were negative following DAA therapy.
- *De novo* HCC developed in three patients (7.5%) post-treatment; all of whom had pre-existing liver cirrhosis.
- Significant changes were observed in liver function tests (ALT, AST, bilirubin, AFP), as well as in markers of liver inflammation and fibrosis, after treatment.
- Platelet counts and liver steatosis showed significant post-treatment changes in most patients following DAA therapy.
- This study is among the first to report residual HCV RNA in bile, gallbladder, and pancreatic tissues post-SVR in an Egyptian patient cohort.
- The study establishes a link between post-treatment HCC and underlying liver cirrhosis, rather than attributing it to DAA treatment itself.

## Graphical abstract


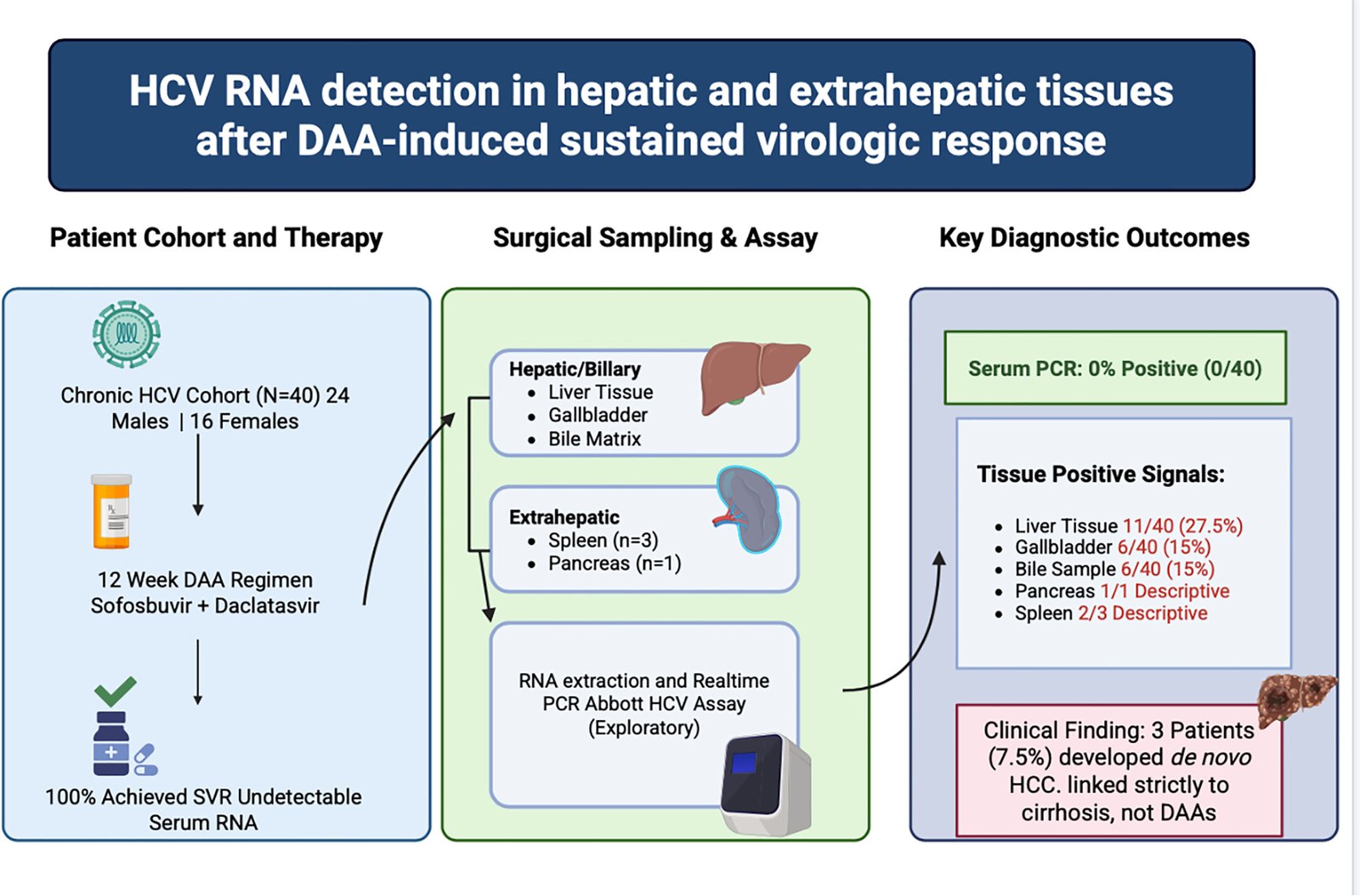


**Graphical abstract summarizing patient flow, surgical sampling, and post-SVR tissue-associated HCV RNA detection.** Forty chronic HCV patients received 12 weeks of sofosbuvir plus daclatasvir and achieved serum PCR-negative SVR. Surgical samples collected 4–12 weeks after SVR confirmation included liver, gallbladder tissue, and bile from all patients, with pancreatic (n = 1) and splenic (n = 3) tissues obtained when clinically available. Despite persistent serum PCR negativity (0/40), HCV RNA signals were detected in liver tissue (11/40, 27.5%), gallbladder tissue (6/40, 15%), bile (6/40, 15%), pancreatic tissue (1/1), and spleen (2/3). Pancreatic and splenic results are descriptive because of small sample size. *De novo* HCC occurred in three cirrhotic patients and was not attributed to DAA therapy or residual RNA. Tissue positivity represents exploratory molecular detection, not confirmed active viral replication. **Abbreviations:** DAA, direct-acting antiviral; HCC, hepatocellular carcinoma; HCV, hepatitis C virus; qRT-PCR, quantitative reverse transcription polymerase chain reaction; SVR, sustained virologic response.

## Data Availability

All data and sources of used materials are available upon request.
